# Mimp/Mtch2, an Obesity Susceptibility Gene, Induces Alteration of Fatty Acid Metabolism in Transgenic Mice

**DOI:** 10.1371/journal.pone.0157850

**Published:** 2016-06-30

**Authors:** Yamit Bar-Lev, Sharon Moshitch-Moshkovitz, Galia Tsarfaty, Dafna Kaufman, Judith Horev, James H. Resau, Ilan Tsarfaty

**Affiliations:** 1 Department of Clinical Microbiology and Immunology, Sackler School of Medicine, Tel Aviv University, Tel Aviv, Israel; 2 Cancer Research Center, Sheba Medical Center, Ramat-Gan, Israel; 3 Department of Diagnostic Imaging, Sheba Medical Center, Ramat-Gan, Israel; 4 Van Andel Research Institute, Grand Rapids, Michigan, 49503, United States of America; Johns Hopkins University School of Medicine, UNITED STATES

## Abstract

**Objective:**

Metabolic dysfunctions, such as fatty liver, obesity and insulin resistance, are among the most common contemporary diseases worldwide, and their prevalence is continuously rising. Mimp/Mtch2 is a mitochondrial carrier protein homologue, which localizes to the mitochondria and induces mitochondrial depolarization. Mimp/Mtch2 single-nucleotide polymorphism is associated with obesity in humans and its loss in mice muscle protects from obesity. Our aim was to study the effects of Mimp/Mtch2 overexpression *in vivo*.

**Methods:**

Transgenic mice overexpressing Mimp/Mtch2-GFP were characterized and monitored for lipid accumulation, weight and blood glucose levels. Transgenic mice liver and kidneys were used for gene expression analysis.

**Results:**

Mimp/Mtch2-GFP transgenic mice express high levels of fatty acid synthase and of β-oxidation genes and develop fatty livers and kidneys. Moreover, high-fat diet–fed Mimp/Mtch2 mice exhibit high blood glucose levels. Our results also show that Mimp/Mtch2 is involved in lipid accumulation and uptake in cells and perhaps in human obesity.

**Conclusions:**

Mimp/Mtch2 alters lipid metabolism and may play a role in the onset of obesity and development of insulin resistance.

## Introduction

Overweight and obesity are a major public health problem, often associated with type 2 diabetes [1, 2], however, the underlying mechanisms are largely unknown [2, 3]. Mimp (Met-induced mitochondrial protein) [4], also designated Mitochondrial Carrier Homolog 2 (Mtch2), encodes a 33-kDa conserved protein that shares sequence and structural homology with members of the mitochondrial carrier protein (MCP) family [4, 5]. Mimp/Mtch2 is localized to the mitochondrial outer membrane and its overexpression significantly decreases mitochondrial membrane potential [4]. Loss of Mimp/Mtch2 in hematopoietic stem cells increases mitochondrial oxidative phosphorylation and drives cells into cell cycle [6]. Mimp/Mtch2 is up-regulated by activated Met, a tyrosine kinase receptor involved in various tumors and by its ligand, hepatocyte growth factor/scatter factor (HGF/SF) [4]. Induction of Mimp/Mtch2 in DA3 mouse mammary carcinoma cells alters Met signaling cascade, its induced motility, invasion *in vitro* and tumor growth *in vivo* [7]. In tumor necrosis factor (TNF)-α-activated FL5.12 cells, Mimp/Mtch2 is a part of a mitochondrial complex with truncated BH3-interacting-domain death agonist (tBID), a proapoptotic BCL-2 family member [5]. tBID modulates lipid oxidation flux and inhibits β-oxidation in apoptotic cells, resulting in the accumulation of lipid metabolites [8]. Mimp/Mtch2 is expressed in a wide variety of tissues, predominantly in liver, kidney, heart, skeletal muscle and testis [4] as well as in human white adipose tissue [1].

Single-nucleotide polymorphism (SNP) in *Mimp/Mtch2* (rs10838738) is associated with high body mass index (BMI) and common obesity in humans [2, 9], increased weight, waist circumference and dietary intake [3, 10] and with decreased insulin sensitivity [11]. Loss of Mimp/Mtch2 in mice muscle increases muscle metabolism and mitochondrial size, and protects mice from diet-induced obesity [12].

In this work we characterize transgenic mice expressing Mimp/Mtch2-GFP. Transgenic Mimp/Mtch2-GFP mice develop fatty livers and kidneys, exhibiting high content of lipid droplets. Maintaining Mimp/Mtch2-GFP mice on high fat diet (HFD) leads to higher blood glucose levels. Mimp/Mtch2-GFP transgenic expression is associated with high levels of acyl-CoA dehydrogenase (MCAD), acetyl-CoA acyltransferase 2 (Thiolase) and fatty acid synthase (FASN), demonstrating alteration of fatty acid metabolism.

Our results demonstrate a new link between Mimp/Mtch2, a mitochondrial carrier homologue, and lipid accumulation in liver and kidney and increased blood glucose levels.

## Methods

### Cells

HEK-293, human embryonic kidney cells [13], and HEK-293T (HEK-293 cells stably transfected with the SV40 large T antigen) [14] were grown in DMEM (GibcoBRL, Gaithersburg, MD) supplemented with 10% fetal calf serum (FCS) (Life Technologies, Gaithersburg, MD). HEK-293 and HEK-293T cells were stably or transiently transfected (respectively) with pMimp/Mtch2-GFP plasmid [4] using calcium phosphate [15].

### Transgenic mice generation

Mimp/Mtch2-GFP sequence under CMV promoter was digested out of pMimp/Mtch2-GFP plasmid [4], purified, and injected into mouse fertilized oocytes of C57BL/6 mice (Jackson Lab). To identify pMimp/Mtch2-GFP transgenic founders, mice were screened by polymerase chain reaction (PCR) analysis of the tail DNA. Mimp/Mtch2-GFP primers: 5′-GGGGTACCATCATGGCGGACGCG-3′/5′-GCGGATCCTGCCCCACATCTTCAAATTA-3′. Mice were maintained in a homozygote breeding and age matched littermates served as controls. In all experiments we used about half males and half female, distributed randomly between the experiments groups.

Mice were maintained in the following conditions: Caging: Thoren individually ventilated cages. Room temperature: 72 degrees F. Light cycle: 12 hours on, 12 hours off. Humidity: 30–70%. Mice are housed on Anderson’s 1/8 in. Bed-O-Cobs. Water is given via an Edstrom automatic watering system. For all experiments mice were anesthetized with 2% isoflurane (Halocarbon Products, River Edge, NJ).

Animal care and experimental procedures were approved by the Van Andel Research Institute and Tel Aviv University Institutional Animal Care and Use Committees, and were conducted in accordance with National Institutes of Health guidelines.

### Mice diet

Mice were maintained on high or low fat diets ad libitum (ad lib) from birth. The high fat diet (HFD) is an industry autoclaved mouse breeder diet (LabDiet #5021), containing 22.3% calories from fat.

The Mimp/Mtch2-GFP mice suffered from low fertility and litter sizes were low. In attempt to increase fertility, mice were transferred to the mouse breeder diet (Labdiet 5021) and as a result developed fatty livers and kidneys. We therefore used this diet as the HFD. Low-fat diet is Teklad Global 18% Protein Rodent Diet, containing 18% calories from fat. Both purchased from Harlan Lab (Jerusalem, Israel).

### Imaging

Intravital and frozen section imaging analyses were carried out using an LSM510 META confocal laser scanning microscope (CLSM) (Zeiss, Jena, Germany) with a lambda unmixing image analysis package (Zeiss, Jena, Germany) [16].

Mitochondrial depolarization evaluation: Cells and organs, stained with JC-1, were imaged in continuous spectra and GFP, JC-1 green and JC-1 red fluorescence signals were separated using lambda unmixing algorithm. To evaluate bleed through, non-transfected HEK-293T cells and cells with GFP, JC-1 or both were measured for the emission of each fluorophore to each detection channel. Image analysis was carried out using MICA image analysis software (Cytoview LTD, Petach Tikva, Israel), as previously described [16].

### Mitochondrial membrane potential analysis

Alterations in mitochondrial membrane potential were assessed using a mitochondrial membrane potential-sensitive dye, JC-1 (Life technologies, Eugene, OR). Cells were stained with 5 μM JC-1 in culture medium for 15 min and exteriorized organs were stained for 30 min. Stained cells and tissues were imaged as described above. To evaluate mitochondrial depolarization in Mimp/Mtch2-GFP expressing cells compared to non-transfected cells, cells were first selected by their Mimp/Mtch2-GFP expression, and then analyzed for JC-1 green and red mitochondrial staining. Non-transfected HEK-293T cells served as control.

### Western blot analysis

Homogenized tissues were lysed with lysis buffer (20mM Tris-HCl, pH = 7.8, 100mM NaCl, 50mM NaF, 1% NP40, 0.1% SDS, 2mM EDTA and 10% glycerol) supplemented with protease inhibitor cocktail (Roche, Mannheim, Germany) and 1mM sodium orthovanadate, separated by SDS-PAGE and transferred onto polyvinylidene difluoride (PVDF) membrane (Invitrogen, Carlsbad, CA, USA). Immuno-detection was performed using mouse anti-GFP (B2) (Santa-Cruz) and mouse anti-actin (Millipore) antibodies.

### Ultrasound Imaging

Ultrasound imaging was performed using 15L8s, 14-MHz linear transducer power Sequoia 512 (Acuson, Mountain View, CA) by an experienced operator who was blinded to laboratory values.

Livers and Kidneys were considered “fatty” when significantly increased echogenicity was observed relative to normal renal parenchyma and normal wild type (WT) mice organs.

### Pathological analyses and staining

Histology and histopathology were characterized using hematoxylin and eosin (H&E) and standard anatomic pathology classifications, by two independent histopathologists. HEK-293T cells and frozen sections of livers and kidneys were fixed for 15 min with 3.7% formaldehyde and 1% calcium chloride in PBS. Samples were incubated in 0.33% Oil red O (ORO) for 15 min at room temperature, washed and imaged with C2020 camera attached to a light microscope IX-50 (Olympus, Tokyo, Japan).

### Blood glucose monitoring

Blood glucose levels of mice, fed on either low or HFD, were monitored weekly. Glucose levels were measured using FreeStyle™ Blood Glucose Monitoring System (Abbott Diabetes Care, Alameda, CA), according to manufacture instructions.

### cDNA microarray analysis

RNA was extracted from: Mimp/Mtch2-GFP transgenic mice (4 mice); normal kidneys (2 mice) and fatty kidneys (2 mice), divided and labeled once with Cy5-dCTP and once with Cy3-dCTP (Perkin Elmer, Boston MA), 8 samples total. Samples were hybridized with a 15,247 mouse cDNAs and then robotically spotted onto polylysine-coated microarray slides (Obtained from the National Institutes on Aging, Baltimore, MD and performed at the Van Andel Research Institute, Michigan). Microarray data are available in the ArrayExpress database (www.ebi.ac.uk/arrayexpress) under accession number E-MTAB-1752. Preliminary processing steps included averaging samples duplicates, removal of unknown genes and duplicates, conversion of all NULL signal (N/A) to zeros and excluding genes with missing signal (N/A). Cluster analysis was performed using the Expander software package [17]. Gene ontology (GO) analysis was performed using WebGestalt, web based bioinformatics analysis tool [18]. Enrichment significance was calculated compared to total array genes using a hypergeometric test.

Gene expression was also analyzed using a previously published cDNA microarray [19] of skeletal muscle obtained from normal-weight (n = 8; BMI 23.8 ± 0.58 kg/m2), overweight/obese (n = 8; BMI 30.2 ± 0.81 kg/m2), and extremely obese (n = 8; BMI 53.8 ± 3.5 kg/m2) females undergoing abdominal surgery [19]. Gene expression profiles of these samples ware later described in [20] and [21].

### Quantitative real-time PCR

Total RNA was isolated using TRI reagent (Biolab, Jerusalem, Israel). cDNA was synthesized using Verso cDNA kit (Thermo scientific, Waltham, MA). mRNA expression was assessed by ABI 9600 HT quantitative real time PCR system (Applied Biosystems, Foster City, CA). Relative quantity (RQ) was normalized compared to the housekeeping gene glyceraldehyde 3-phosphate dehydrogenase (GAPDH). RNA from HEK-293 cells was also checked compared to hypoxanthine phosphoribosyl transferase (HPRT1).

The primers used in the real-time PCR: GAPDH 5'-TGCACCACCAACTGCTTAGC-3'/5'-GGCATGGACTGTGGTCATGAG-3', HPRT1 5'-ATGGACTGATTATGGACAGGACTG-3'/5'-TCCAGTAGGTCAGCAAAGAAC-3', Mimp/Mtch2 5'-GGAGTCTGAGAAACCTGAGGAGT-3'/5'-CAGCAGAACGAGCAATCATC-3', MCAD 5'-TGTCGAACACAACACTCGAAA-3'/5'-CTGCTGTTCCGTCAACTCAA-3', Thiolase 5'-CCCTGCTATCAATGGAGCAT-3'/5'-AGAACTGAGGGGCAAAAGC-3', FASN 5'-GGAGGTGGTGATAGCCGGTAT-3'/5'-TGGGTAATCCATAGAGCCCAG-3', hMCAD 5'-AGGAGCCATTGATGTGTGC-3'/5'-CTGCTTTGGTCTTTATACCAGCTA-3', hThiolase 5'-CAAACCACCCTGGAACAGTTA-3'/5'-TCCAGCACCATCAGCTACAC-3', hFASN 5'-GTTCACGGACATGGAGCAC-3'/5'-GTGGCTCTTGATGATCAGGTC-3'.

### Statistical analysis

All data are expressed as means ± standard error of the mean (SEM). Significant differences were assessed by a two-tailed Student's t-test, one-way ANOVA with Tukey’s post hoc or chi-square test.

## Results

### Mimp/Mtch2-GFP expression and localization in transgenic mice

**Fig 1:**
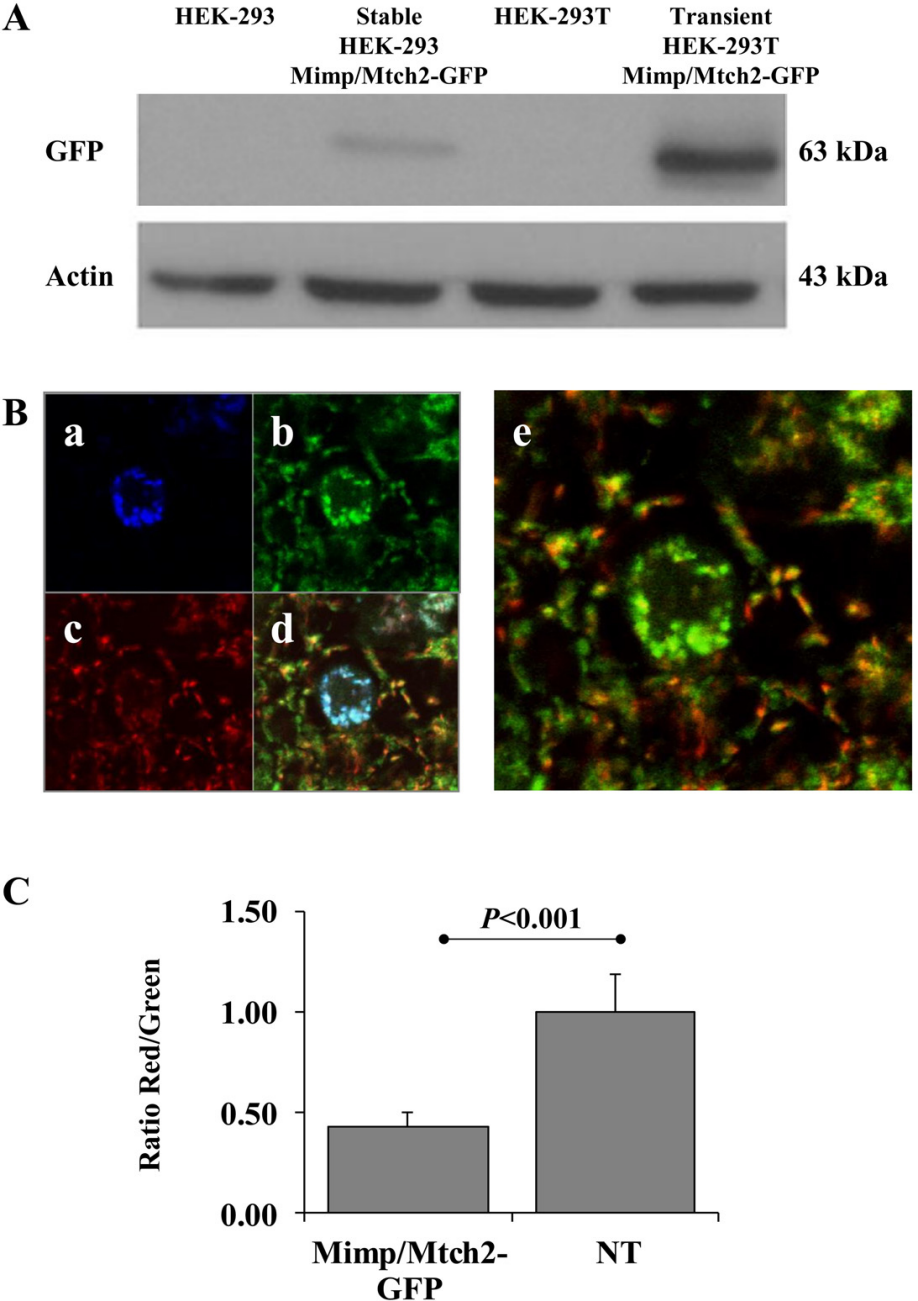
Mimp/Mtch2-GFP expression in HEK-293 and in HEK-293T cells. **A.** Western blot of control non-transfected HEK-293 cells, stable HEK-293 Mimp/Mtch2-GFP, non-transfected HEK-293T cells and from HEK-293T cells transiently transfected with Mimp/Mtch2-GFP. Western blot was performed using antibody specific for GFP, which recognizes Mimp/Mtch2-GFP expression (64kDa). Total cell actin served as internal control. **B.** Mitochondrial Membrane potential of HEK-293T cells transfected with Mimp/Mtch2-GFP and stained with JC-1. Images of each fluorescent emission are represented in (a) Mimp/Mtch2-GFP, (b) JC-1 green, (c) JC-1 red and (d) the overall overlay. (e) Overlay of only the JC-1 Green and Red staining. **C.** Alteration in the mitochondrial potential. The normalized R/G fluorescence ratio was calculated only for cells expressing Mimp/Mtch2-GFP (n = 50, *P* = 3.6 x 10^−8^, Student's t-test). “NT” represents non-transfected cells, served as control. Results are an average of 5 independent experiments and expressed as means ± SEM.

Mimp/Mtch2-GFP is localized to the mitochondria [4]. To assess its functionality, we monitored its effect on the mitochondrial membrane potential, using JC-1. To differentiate GFP and JC-1 signals we used spectral CLSM-analysis, in which specific fluorescent signals of GFP, JC-1 green and JC-1 red, are unmixed (see methods). HEK-293T cells, transiently expressing Mimp/Mtch2-GFP (Fig 1A) exhibited lower red to green (R/G) fluorescence ratio (Fig 1B and 1C). These results demonstrate that Mimp/Mtch2-GFP retains its activity as it induces substantial depolarization of the mitochondrial potential, in a manner similar to the native Mimp/Mtch2 [4].

To explore the in vivo effects of Mimp/Mtch2-GFP we generated transgenic mice overexpressing it. Mimp/Mtch2-GFP transgenic mouse line suffered from low fertility rate and was hard to maintain. Mimp/Mtch2-GFP average litter size was 2.9±1.4 as compare to 6.7 pups/litter in WT mice [22], and the last generations had no progeny. This could indicate a role for Mimp/Mtch2 in reproductively.

**Fig 2:**
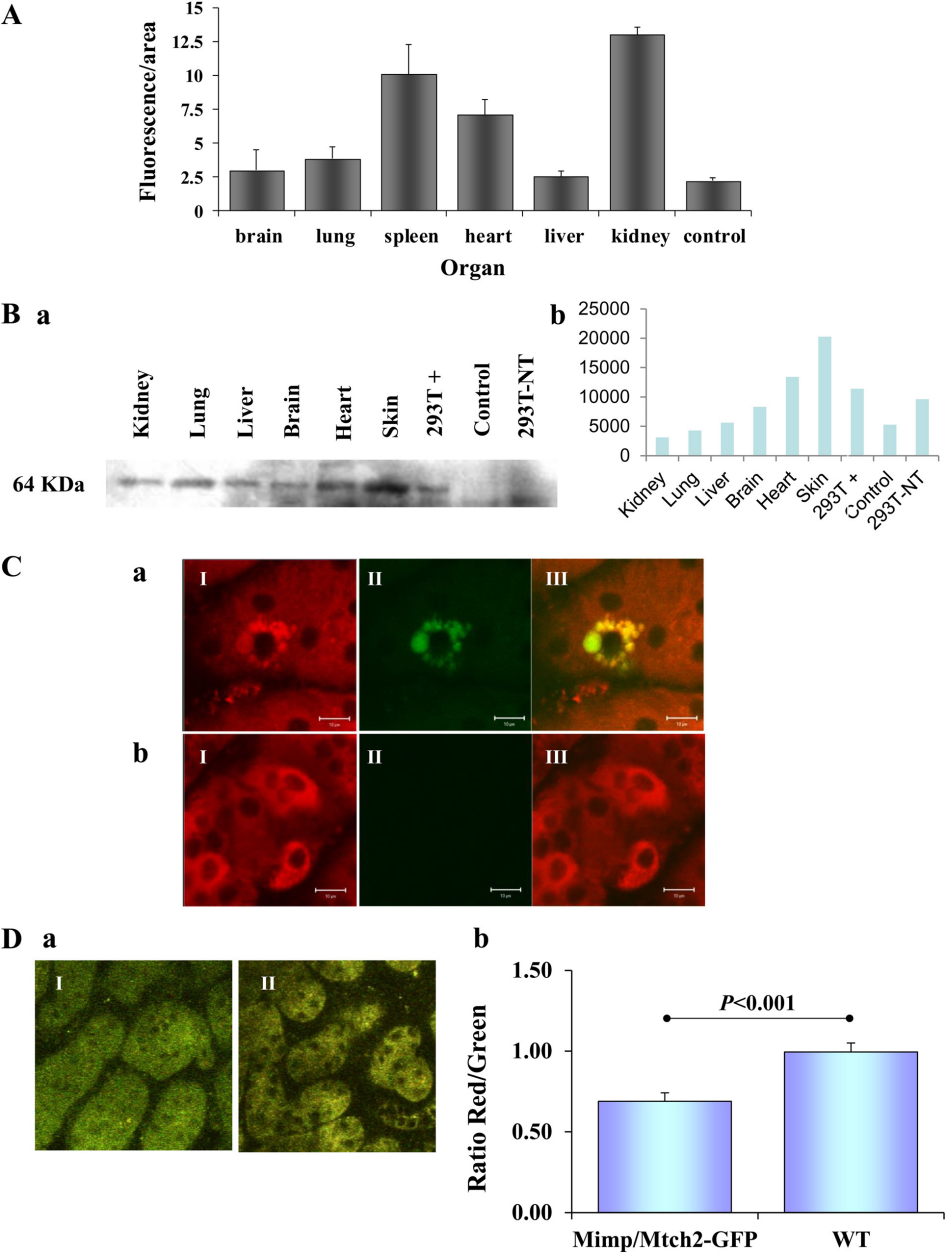
Characterization of Mimp/Mtch2-GFP Expression in Transgenic Mice. **A.** Quantitative analysis of the fluorescent signal from CLSM images of exteriorized Mimp/Mtch2-GFP transgenic mice tissues (n = 6) and wild type (WT) mice kidney (n = 2), performed by MICA software. **B.** (a) Western blot analysis of Mimp/Mtch2-GFP levels in tissue lysates from transgenic mice organs. “+” represents HEK-293T cells transiently transfected with Mimp/Mtch2-GFP as a positive control; “Control” represents lysate obtained from control, non-transgenic mouse kidney; “N.T.” represents non-transfected cells. (b) Quantitative analysis of a representative western blot (presented in “a”), performed using Image J software. **C.** Mimp/Mtch2-GFP localization in mitochondria of exteriorized kidneys. (I) Red Mitotracker staining of the mitochondria. (II) GFP fluorescence. (III) Overlay showing co-localization of the two signals in the mitochondria. (a) Mimp/Mtch2-GFP transgenic mice. (b) WT mice. **D.** Mitochondrial membrane potential of transgenic mice kidney. (a) Images of excised kidneys stained *in vivo* with JC-1 and imaged 15 min after staining. (I) Mimp/Mtch2-GFP. (II) WT. (b) Quantification of the signals and the Red/Green ratios obtained from excised kidneys of WT (n = 7) and Mimp/Mtch2-GFP (n = 9) mice, compared by Student's t-test. All bar graphs results are expressed as means ± SEM. All Mice were 14 month old.

Transgenic expression of Mimp/Mtch2-GFP, as measured by GFP fluorescence using CLSM analysis, varied significantly between the different organs, exhibiting higher expression levels in the heart, kidney and spleen (Fig 2A). Organs of control mice showed very low fluorescence background. The presence of Mimp/Mtch2-GFP was further validated using western blot analysis of tissue lysates (Fig 2B), however, GFP amount in the different organs was different from the fluorescent results, probably due to differences in methods sensitivity. Next, we confirmed the mitochondrial localization of Mimp/Mtch2-GFP in vivo in intact kidneys that demonstrate co-localization of the GFP and Mitotracker signals (Fig 2C). These results show that Mimp/Mtch2-GFP is expressed in a wide range of mouse tissues and that it is properly localized to the mitochondria.

### Mimp/Mtch2-GFP induces depolarization of the mitochondrial membrane potential in vivo

To study Mimp/Mtch2-GFP effect on mitochondrial membrane potential *in vivo*, mice were injected with JC-1 (Fig 2Da). Non-transgenic mice served as controls. We assessed R/G fluorescence ratios in the kidneys, only for cells expressing Mimp/Mtch2-GFP. Normalized R/G fluorescence ratio revealed mitochondrial depolarization of Mimp/Mtch2-GFP transgenic kidney compared to non-transgenic kidneys (Fig 2Db). These results indicate that Mimp/Mtch2-GFP induces depolarization of the mitochondrial potential *in vivo*.

### Mimp/Mtch2-GFP mice develop fatty liver and kidney

**Fig 3:**
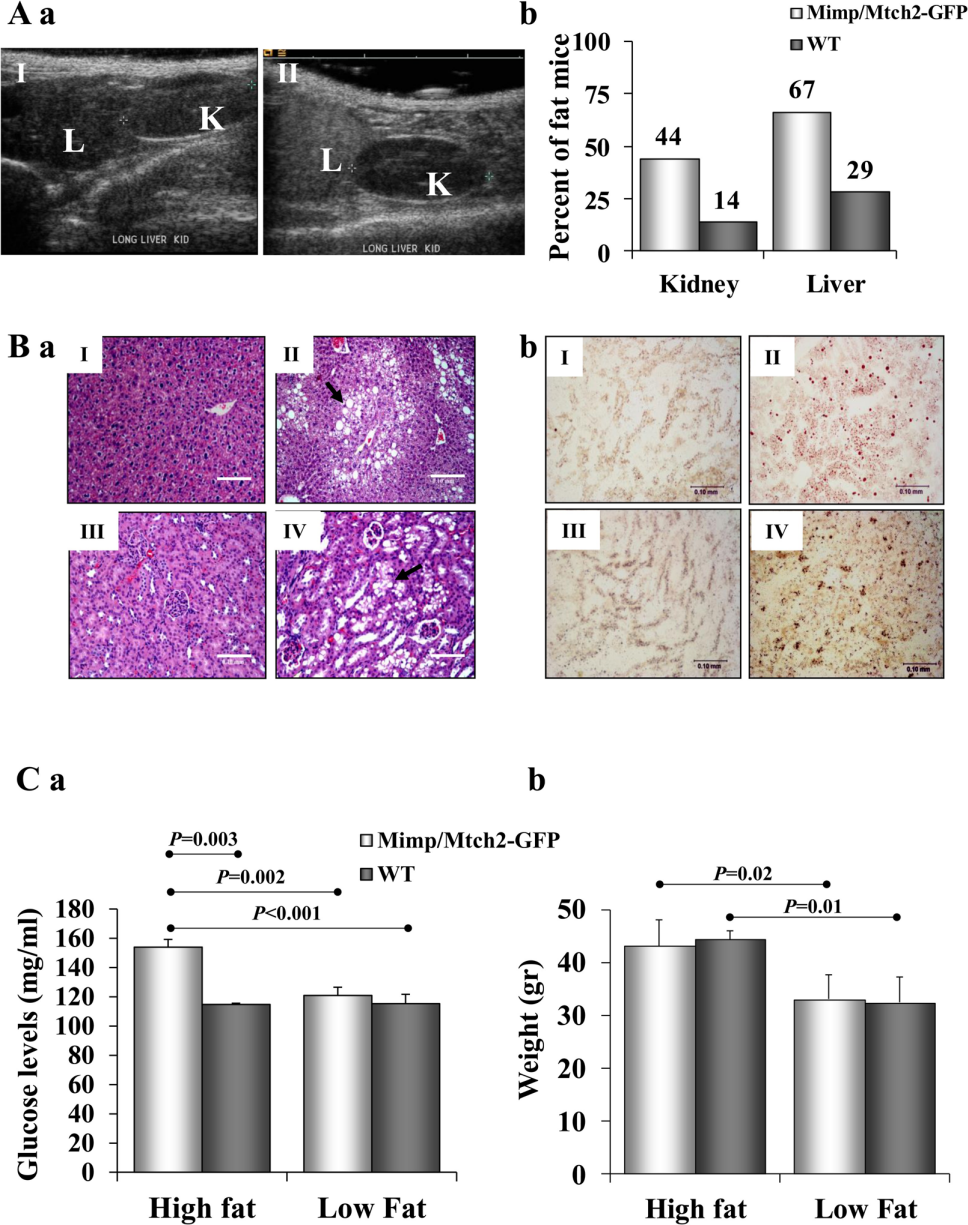
Detection of fatty livers and kidneys and blood glucose levels in Mimp/Mtch2-GFP transgenic mice. **A.** (a) Ultrasound analysis of control (I) and Mimp/Mtch2-GFP transgenic mouse (II), 14 month old. (b) Calculation of % of mice showing fatty changes in H&E stained fixed sections from Mimp/Mtch2-GFP (n = 18) and WT (n = 7) mice. *P* = 0.0003 for both liver and kidney (Chi test). All mice were maintained on HFD. **B.** (a) H&E staining of fixed sections. (I) Control mouse liver. (II) Liver section of Mimp/Mtch2-GFP transgenic mouse. Fat vesicles are indicated by arrow. (III) Control mouse showing a normal kidney. (IV) Kidney section obtained from a Mimp/Mtch2-GFP transgenic mouse shows fatty vesicles that accumulate mainly in the proximal convoluted tubules of the kidney (indicated by arrow). Bars represent 0.10 mm. (b) Oil-Red-O staining of Mimp/Mtch2-GFP transgenic mouse showing fatty liver (II) and fatty kidney (IV), and of control mouse showing normal liver (I) and kidney (III). All mice were between the ages of 12 and 14 month and maintained on HFD from birth. **C.** Glucose levels and weight in Mimp/Mtch2-GFP mice. Mimp/Mtch2-GFP transgenic mice (n = 16) and age matched control mice (n = 12) consuming high or low fat diets were monitored weekly in the mornings for their (a) blood glucose levels and (b) their weight. Measurements were performed between the ages of 9 to 16 month and then averaged for all time points and all mice. All bar graphs results are expressed as means ± SEM. Groups were compared using ANOVA with Tukey’s post hoc.

An ultrasound follow-up of 12-month-old Mimp/Mtch2-GFP transgenic mice consuming HFD revealed that mice tend to develop fatty livers (Fig 3Aa II, right), relative to control age-matched mice from the same genetic background (Fig 3Aa I, left). H&E staining, performed on 18 Mimp/Mtch2-GFP and 7 WT mice, revealed that 67% of Mimp/Mtch2-GFP transgenic mice develop fatty livers (Fig 3Ba II, upper right), compared to only 29% of control mice (Fig 3Ba I, upper left, *P* = 0.0003). These rates were further confirmed by ORO staining (Fig 3Bb) of liver sections. In addition to lipid accumulation, fatty livers also showed accumulation of glycogen and water vesicles that was confirmed by periodic acid-schiff (PAS) staining (data not shown).

Forty four percent of Mimp/Mtch2-GFP-transgenic mice consuming HFD also developed fatty kidneys, compared to 14% of age-matched control mice (Fig 3Ab, *P* = 0.0003). Fat vesicles in the kidneys appeared to accumulate mainly in the proximal convoluted tubules (Fig 3B IV, lower right), while vesicles containing glycogen and proteinaceous materials were evident mainly in ductal tubes of the medulla. PAS staining further validated these observations (data not shown). Fat vesicles were stained positive for Oil-red-O (Fig 3Bb) and were absent in sections of normal control kidneys (Fig 3Ba III and 3Bb III, left). Mice maintained on low fat diet did not develop fatty liver and kidney (data not shown).

Our results demonstrate that Mimp/Mtch2 overexpression leads to fat accumulation in kidneys and livers in mice consuming HFD.

### Mimp/Mtch2-GFP leads to alteration in blood glucose levels

Mimp/Mtch2-GFP mice maintained on either low or HFD from birth were monitored weekly from the age of 9 month to 16 month for blood glucose levels and weight. Mice consuming HFD gained more weight than mice consuming low fat diet, however, no significant difference was detected between Mimp/Mtch2-GFP and WT mice consuming each of the diets (Fig 3Cb). Significant increase was observed in blood glucose levels in Mimp/Mtch2-GFP transgenic mice maintained on HFD (154mg/ml) compared to low fat diet (115mg/ml) (Fig 3Ca, *P* = 0.002), and compared to control mice consuming either diet (ANOVA, Tukey’s post hoc). Our results show that *in vivo* overexpression of Mimp/Mtch2-GFP in mice consuming HFD leads to fat accumulation in the kidney and liver as well as to increased glucose levels.

### Gene expression profiling during fat accumulation in transgenic mice

**Fig 4:**
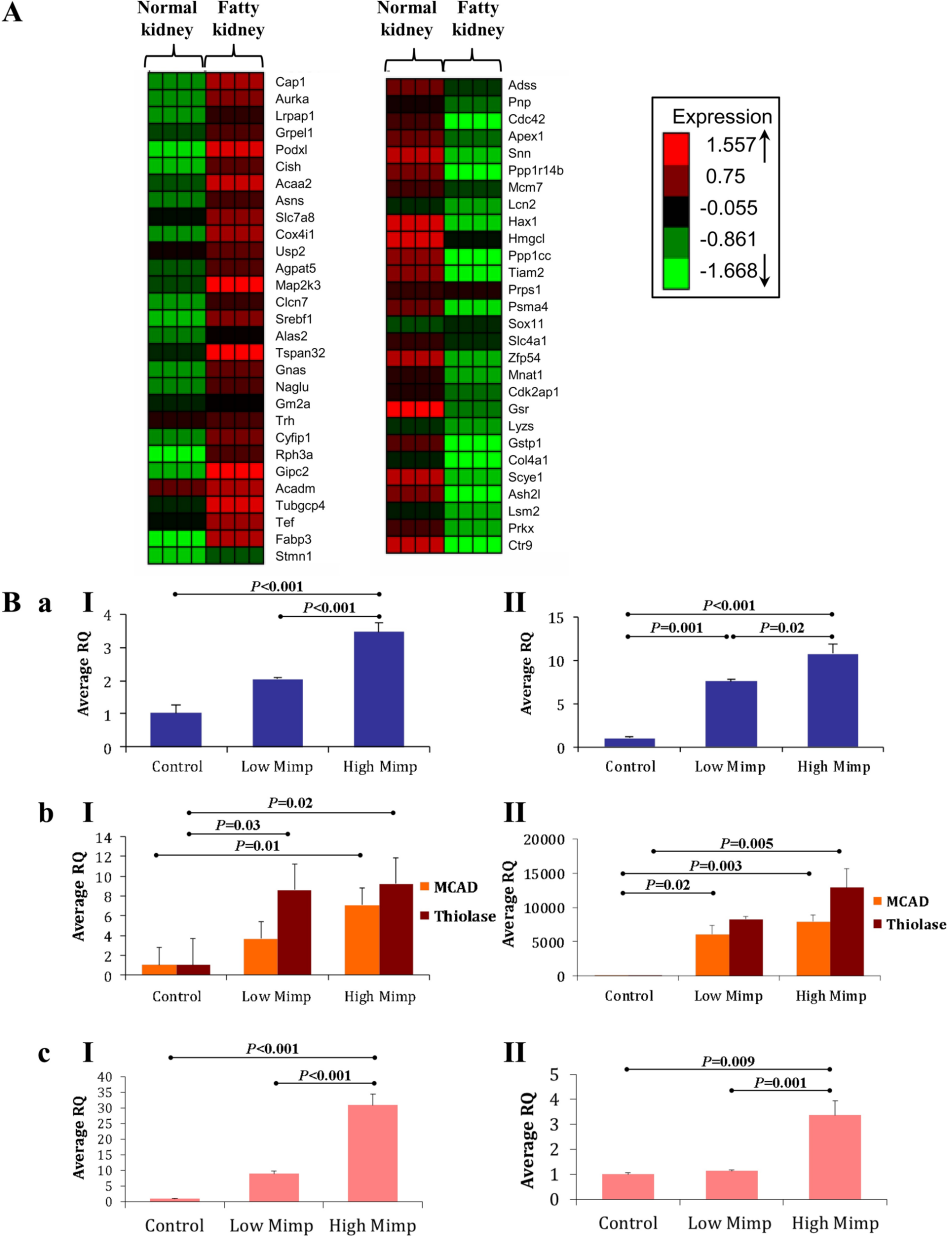
Cluster analysis of cDNA microarray and gene expression in Mimp/Mtch2-GFP mice. **A.** K-Means clustering over 57 probes that change significantly (p<0.05) in fatty (n = 2) compared to non-fatty (n = 2) kidneys of Mimp/Mtch2-GFP transgenic mice consuming HFD. Rows, genes; Columns, samples. Down-regulation is colored in green and up-regulation is colored in red. **B.** Quantitative real time PCR (qRT-PCR) for gene expression of (a) Mimp/Mtch2, (b) MCAD and Thiolase and (c) FASN, in livers (I) and kidneys (II) of control (WT) and Mimp/Mtch2-GFP transgenic mice, in the ages of 21–23 month, consuming low fat diet. The transgenic mice were divided into 2 groups of low and high Mimp/Mtch2 according to Mimp/Mtch2 expression levels. Each group consists of at least 3 mice. Results are represented as averaged relative quantity (RQ) of the mice in each group. All bar graphs results are expressed as means ± SEM. Groups were compared using ANOVA with Tukey’s post hoc.

To obtain a comprehensive picture of changes in gene expression along the process of fat accumulation in our transgenic mice, kidney samples from 4 different Mimp/Mtch2-GFP transgenic mice consuming HFD, two samples of normal kidneys and two of fatty kidneys, were subjected to cDNA microarray analysis. Only 57 genes exhibited significant differential expression between normal and fatty kidneys (p<0.05). Differential gene expression analysis revealed two major clusters (Fig 4A), each showing an overall homogeneity of 0.8, which reflects a high similarity between genes assigned to each cluster. Significantly enriched functional annotations in each cluster were analyzed using the WebGestalt tool (*P*<0.05).

#### Cluster 1

(Fig 4Aa) consists of 29 up-regulated genes. Biological process annotations of these genes showed highly significant enrichment for lipid metabolism genes (P = 0.0002). Among these genes are enzymes involved in membrane lipid metabolism (*Agpat5*, *Gm2a* and *Fabp3*), fatty acid metabolism (*Acadm* and *Acaa2*) and *Srebf*1, a sterol regulatory element-binding transcription factor 1 that regulates genes required for synthesis and uptake of cholesterol, fatty acids, triglycerides, and phospholipids (19, 21). Enrichment was also found for protein binding (13 genes, P = 0.03) and intracellular signaling (5 genes, P = 0.04) genes (Table A in S1 File).

#### Cluster 2

(Fig 4Ab) consists of 28 down-regulated genes. Functional annotation analysis of the down-regulated genes demonstrated significant enrichment of mitochondrial proteins (5 genes, *P* = 0.004), 2 of them are mitochondrial membrane genes (*Ppp1cc* and *Hmgcl*). Genes involved in cellular metabolism were significantly enriched as well (16 genes, *P* = 0.04), these included genes associated with nucleic acid metabolism (10 genes, *P* = 0.02) and glutathione metabolism (2 genes, *P* = 0.0004). Among additional physiological process enriched in this cluster we found 4 genes involved in cell cycle (*P* = 0.02) and 2 genes involved in cell division (*P* = 0.04) (Table B in S1 File).

We focused on the 2 genes involved in fatty acid metabolism and up-regulated in fatty kidneys, Acyl-Coenzyme A dehydrogenase (Acadm), also called Medium chain Acyl-Co A dehydrogenase (MCAD), and Acetyl-Coenzyme A acyltransferase 2 (Acaa2), also named 3-Ketoacyl-CoA thiolase (Thiolase). MCAD catalyzes the first step of each cycle of mitochondrial fatty acid β-oxidation [23, 24], and Thiolase catalyzes the last step [24]. These 2 genes may, therefore, play a role in Mimp/Mtch2-induced lipid accumulation.

To validate and examine the expression of MCAD and Thiolase in transgenic mice compared to WT mice, RNA was extracted from livers and kidneys of 6 transgenic mice and 2 WT mice and subjected to quantitative real-time PCR. To evaluate the effect of Mimp/Mtch2 on gene expression, regardless of fat accumulation, mice were maintained on low fat diet. Transgenic mice were divided into 2 groups of low and high Mimp/Mtch2 according to averaged Mimp/Mtch2 mRNA expression levels (Fig 4Ba). The expression levels of MCAD, Thiolase and FASN, the only mammalian protein capable of *de novo* fatty acid synthesis [25] were monitored.

MCAD and Thiolase were highly expressed in both livers and kidneys of transgenic mice expressing high Mimp/Mtch2 relative to WT mice. The expression of Thiolase in the liver and MCAD in the kidney was also significantly higher in low Mimp/Mtch2 expression group compared to WT mice (*P* = 0.03 Fig 4Bb II and *P* = 0.02 Fig 4Bb II respectively). Expression levels of FASN in the liver and kidney were significantly higher in the high Mimp/Mtch2-GFP group compared to both WT and low Mimp/Mtch2-GFP mice groups (Fig 4Bc). These results show that transgenic expression of Mimp/Mtch2-GFP in mice increases the expression of MCAD, Thiolase and also of FASN.

### Mimp/Mtch2 increases fatty acid metabolism-related genes and fatty acid uptake in vitro

**Fig 5:**
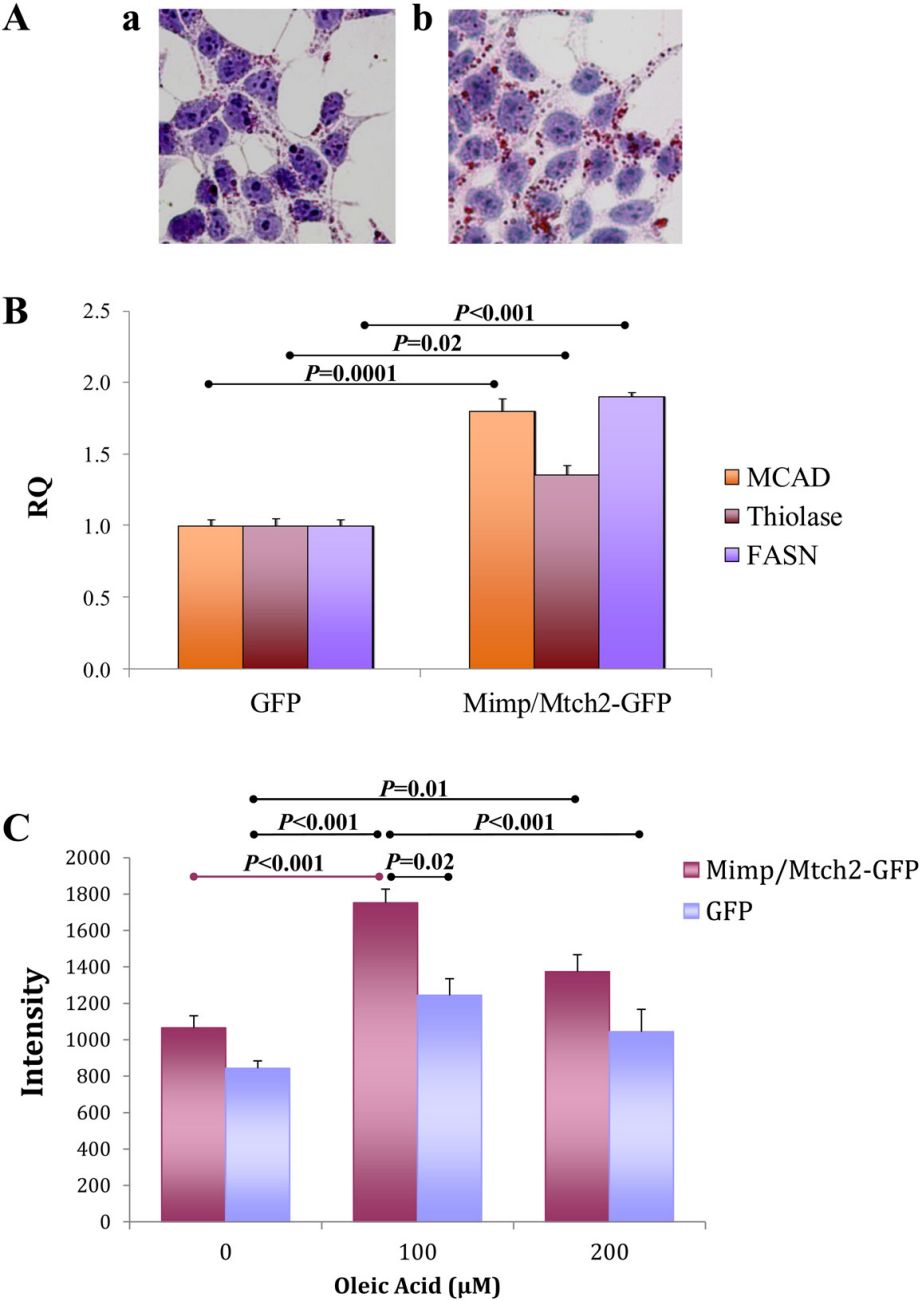
Mimp/Mtch2 increases expression of lipids, lipid uptake and lipid metabolism genes in HEK-293T cells. **A.** Oil-red-O staining of HEK-293T cells transiently expressing Mimp/Mtch2-GFP (b) and control non-transfected cells (a). **B.** HEK-293T cells were transfected with Mimp/Mtch2-GFP or an empty GFP vector. RNA was extracted and measured by qRT-PCR for the expression of MCAD, Thiolase and FASN. Expression between the groups was compared using Student’s t-test. **C.** HEK-293T cells were transfected with Mimp/Mtch2-GFP or an empty GFP vector. Cells medium was supplemented with 100 or 200 μM oleic acid for 24 hours and cells were stained for triglycerides using oil-red-O. Cells were imaged using Leica and oil-red-O signal intensity was quantified using Image J software. Each bar represents an average of at least 6 fields and experiment was repeated 3 times. Groups were compared using ANOVA with Tukey’s post hoc. All bar graphs results are expressed as means ± SEM.

To test whether Mimp/Mtch2-GFP expression also leads to fat accumulation in vitro, we examined its effect in HEK-293T embryonic kidney cells. Transient overexpression of Mimp/Mtch2-GFP in HEK-293T cells revealed accumulation of vesicles stained positively for ORO (Fig 5Ab). This supports a role for Mimp/Mtch2 in lipid metabolism.

Next, we examined Mimp/Mtch2-GFP effect on the expression of MCAD, Thiolase and FASN in these cells. Overexpression of Mimp/Mtch2-GFP leads to increased expression of MCAD (p = 0.0001), Thiolase (p = 0.02) and FASN (p<0.001) (Fig 5B).

We also examined the effect of Mimp/Mtch2-GFP overexpression on fatty acid uptake in HEK-293T cells. Transfected cells only were tested for triglycerides after administration of oleic acid, a long-chain fatty acid, to the growth medium. In cells transfected with GFP alone, oleic acid did not increase lipid accumulation significantly. In cells expressing Mimp/Mtch2-GFP, administration of oleic acid in concentration of 100 μM led to an increase in the amount of lipid vacuoles (p<0.001) and this lipid accumulation was significantly higher than in control cells treated with 100 μM oleic acid (1.3 Fold, p = 0.02, ANOVA) (Fig 5C). These results indicate that Mimp/Mtch2 increases the uptake of fatty acids into HEK-293T cells.

### Bioinformatics analysis of Mimp/Mtch2 role in obesity in humans

**Fig 6:**
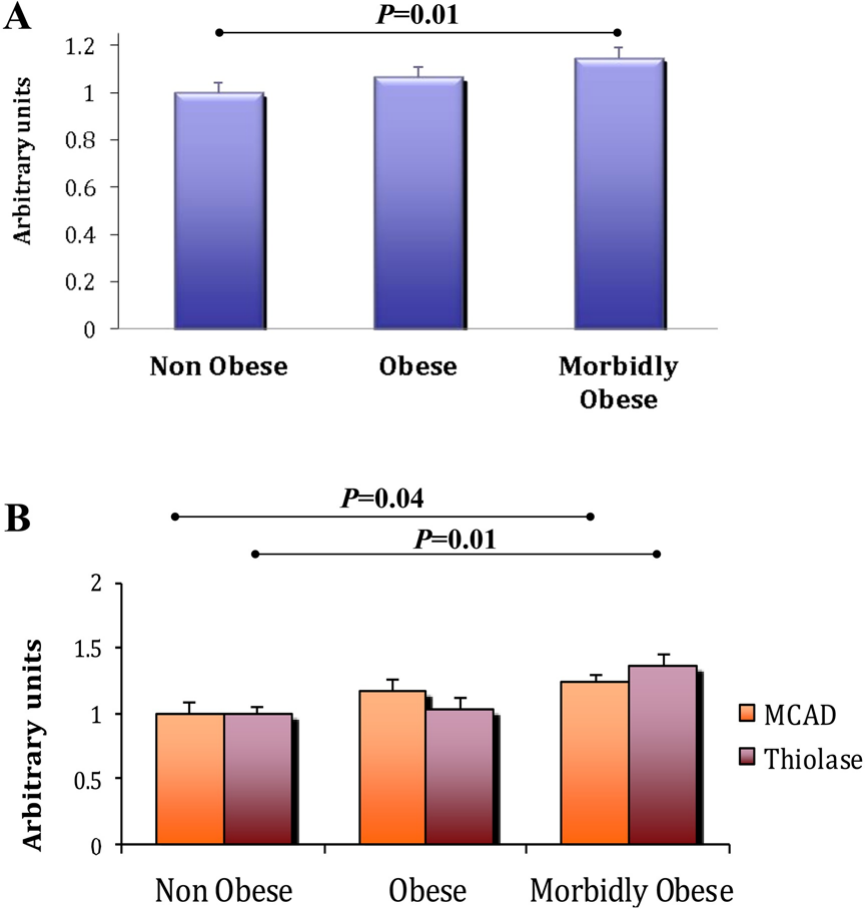
Gene expression in obesity. The expression levels of: **A.** Mimp/Mtch2 and **B.** MCAD and Thiolase, in pre-published cDNA microarray from human skeletal muscle of non-obese, obese and morbidly obese patients [19]. All bar graphs results are expressed as means ± SEM. The non-obese and morbidly obese groups were compared using Student’s t-test.

Our results demonstrate a strong positive link between Mimp/Mtch2 expression and fat accumulation both in vitro and in vivo. In addition, genome-wide association and meta-analysis studies have identified variants at loci in mimp/mtch2 as associated with BMI and common obesity in humans [2, 9]. This prompted us to examine the expression of Mimp/Mtch2 and its related genes in obesity. We analyzed Mimp/Mtch2 mRNA levels using pre-published cDNA microarray [19] of skeletal muscle derived from normal-weight (n = 8; BMI 23.8±0.58 kg/m2), overweight/obese (n = 8; BMI 30.2±0.81 kg/m2), and extremely obese (n = 8; BMI 53.8±3.5 kg/m2) females undergoing abdominal surgery [19]. In these patients, total intramyocellular lipid content in the skeletal muscle was not different between the normal-weight and obese patients, but was high in morbidly obese compared with the normal and obese patients [19]. Mimp/Mtch2 mRNA levels increased non-significantly in obese patients and significantly in morbidly obese patients (P = 0.01) (Fig 6A). The expression levels of both MCAD and Thiolase are also increased significantly in morbidly obese patients (P = 0.04 and P = 0.01 respectively) (Fig 6B). These results demonstrate a positive correlation between human fat accumulation and Mimp/Mtch2 expression levels, which may lead to the increased MCAD and Thiolase levels we observed.

## Discussion

In this work we show that Mimp/Mtch2 overexpression induces fat accumulation in livers and kidneys in mice consuming HFD. Fat accumulation in the liver was recently associated with increased expression of uncoupling protein-2 (UCP-2), another member of the MCP family. UCP-2 overexpression leads to decreased mitochondrial membrane potential and to ATP depletion, associated with obesity-induced fatty liver [26, 27].

Although fatty kidney is less documented, renal lipid accumulation, lipotoxicity, has been reported in animal models of obesity and in metabolic syndrome [28–30]; Obese mice consuming HFD develop lipid accumulation in the glomeruli and proximal tubules as well as increased oxidative stress compared to low-fat diet feeding mice [31]. Lipid accumulation in the renal cortex was also reported in Zucker diabetic fatty rats consuming HFD [32].

Development of fatty livers and kidneys in Mimp/Mtch2-GFP transgenic mice may shed light on key enzymes in fatty organ generation. These mice express higher levels of MCAD and Thiolase, which are further up-regulated in Mimp/Mtch2-GFP mice fatty compared to non-fatty kidneys. MCAD and Thiolase catalyze the first and last steps of mitochondrial fatty acid β-oxidation [23, 24], raising the question of why genes involved in fatty acid catabolism are increased during processes of fat accumulation.

In patients with non-alcoholic steatohepatitis (NASH), increased hepatic lipid accumulation is compensated by increased mitochondrial β-oxidation [33, 34]. Mitochondrial β-oxidation is also enhanced in the liver of genetically obese-diabetic mice with massive steatosis [35, 36]. Significant enrichment of enzymes involved in β-oxidation and lipogenesis was also evident in livers of obese compared to lean mice [37]. These data implicates that the increased expression of MCAD and Thiolase, observed when Mimp/Mtch2 is overexpressed, may serve as a compensatory adaptation to the increased lipid accumulation.

Gene expression profiles of Mimp/Mtch2-GFP transgenic mice having fatty kidneys revealed up-regulation of genes involved in cell signaling and down-regulation of genes involved in cell cycle and cell division. This is in alignment with previous publications showing that Mimp/Mtch2 affects Met-HGF/SF signaling proteins and leads to cell cycle arrest [7]. In hematopoietic cells loss of Mimp/Mtch2 drives the cells into cell cycle [6]. Down-regulated genes were enriched with mitochondrial genes, which may be related to Mimp/Mtch2 yet unknown mitochondrial function.

Our results also demonstrate that FASN, which regulates *de novo* fatty acid synthesis [25], is highly expressed in livers and kidneys of Mimp/Mtch2-GFP mice compared to WT. Previous reports demonstrated that FASN, and its main transcriptional regulator, sterol regulatory element-binding protein (SREBP1) [38], are major players in lipid accumulation in both liver and kidney [39–43]. In the liver, SREBP1 overexpression induces fat accumulation [40, 44]. In the kidney, HFD increases renal lipid accumulation and mRNA expression levels of SREBP1c, FASN, and acetyl-CoA carboxylase (ACC) [42, 43]. Interestingly, higher SREBP1 (encoded by the SREBF1 gene) expression was observed in fatty kidneys of Mimp/Mtch2-GFP transgenic mice on HFD, further supporting a role for Mimp/Mtch2 overexpression in lipid accumulation.

FASN, SREBP1, MCAD and Thiolase may, therefore, be candidate proteins playing major roles in the molecular mechanisms of Mimp/Mtch2-induced lipid accumulation.

Fatty liver and kidney are mainly associated with obesity, type 2 diabetes and the metabolic syndrome [28–30, 45]. Indeed we show here that Mimp/Mtch2 is highly expressed in skeletal muscle of human morbidly obese patients compared to non-obese patients along with increased expression of MCAD and Thiolase.

Although Mimp/Mtch2-GFP transgenic mice are not obese, evidence from cell lines and animal models supports the presence of obesity-related increases in renal and liver lipid accumulation. It was demonstrated that *Mimp/Mtch2* is highly expressed in human adipocytes, with increased levels in obese compared to lean women [1], as well as in the subcutaneous and mesenterial fat of Zucker diabetic obese rats as compare to lean rats [46]. It is possible that the fatty liver and kidney observed in Mimp/Mtch2-GFP transgenic mice may represent an early step towards obesity.

We show that Mimp/Mtch2-GFP transgenic mice consuming HFD exhibit higher blood glucose levels compared to control mice fed with the same diet. The involvement of Mimp/Mtch2 in diabetes is supported by a SNP in *Mimp/Mtch2* gene which is correlated with increased insulin resistance [11] and with increased cardiovascular disease risk in women with preexisting type 2 diabetes [10].

Recent publication shows that Mimp/Mtch2 loss in muscle leads to altered mitochondrial metabolism and protects from diet induced obesity and from hyperinsulinemia [12]. Our results demonstrate a new link between Mimp/Mtch2, a mitochondrial protein, and lipid accumulation processes. Our model suggests that Mimp/Mtch2 leads to mitochondrial functional dysregulation, thus leading to altered metabolic phenotypes, along with gene expression profile compatible with such metabolic alterations. We propose that by inducing the expression of FASN, SREBP1, MCAD and Thiolase, Mimp/Mtch2 increases lipid accumulation, fatty liver and kidney and high blood glucose levels in the transgenic mice. Features of the metabolic syndrome include central adiposity, increased triglycerides and free fatty acids, hyperglycemia, insulin resistance and increased inflammation and hypertension [47]. Several of these features are influenced by Mimp/Mtch2, tempting us to speculate that Mimp/Mtch2 plays a role in the onset and development of metabolic syndrome.

## Supporting Information

S1 File Gene ontology of differentially expressed genes in cDNA microarray of fatty compared to non-fatty kidney of Mimp/Mtch2-GFP transgenic mice. Table A. Up-regulated genes. Table B. Down-regulated genes.
(DOCX)
