# Supplementary material for: Mimp/Mtch2, an Obesity Susceptibility Gene, Induces Alteration of Fatty Acid Metabolism in Transgenic Mice
Source: PLoS One. 2016 Jun 30;11(6):e0157850. doi: 10.1371/journal.pone.0157850 (PMC4928869; doi:10.1371/journal.pone.0157850)
Supplement: S1 File — Table A. Up-regulated genes. Table B. Down-regulated genes. (DOCX) [file pone.0157850.s001.docx]

**Supporting information**

**Table A**

| ***Symbol*** | **Name** |
| --- | --- |
| ***Lipid metabolism*** | |
| *Agpat5* | 1-acylglycerol-3-phosphate O-acyltransferase 5 |
| *Acadm* | acyl-Coenzyme A dehydrogenase, medium chain |
| *Srebf1* | sterol regulatory element binding factor 1 |
| *Acaa2* | acetyl-Coenzyme A acyltransferase 2 (mitochondrial 3-oxoacyl-Coenzyme A thiolase) |
| *Gm2a* | GM2 ganglioside activator protein |
| *Fabp3* | fatty acid binding protein 3, muscle and heart |
| ***Fatty acid metabolism*** | |
| *Acadm* | acyl-Coenzyme A dehydrogenase, medium chain |
| *Acaa2* | acetyl-Coenzyme A acyltransferase 2 (mitochondrial 3-oxoacyl-Coenzyme A thiolase) |
| ***Membrane lipid metabolism*** | |
| *Agpat5* | 1-acylglycerol-3-phosphate O-acyltransferase 5 |
| *Gm2a* | GM2 ganglioside activator protein |
| *Fabp3* | fatty acid binding protein 3, muscle and heart |
| ***Intracellular signaling*** | |
| *Map2k3* | mitogen activated protein kinase kinase 3 |
| *Stmn1* | stathmin 1 |
| *Trh* | thyrotropin releasing hormone |
| *Cish* | cytokine inducible SH2-containing protein |
| *Gnas* | GNAS (guanine nucleotide binding protein, alpha stimulating) complex locus |
| ***Cell morphogenesis*** | |
| *Cyfip1* | cytoplasmic FMR1 interacting protein 1 |
| *Cap1* | CAP, adenylate cyclase-associated protein 1 (yeast) |
| *Stmn1* | stathmin 1 |
| *Cish* | cytokine inducible SH2-containing protein |
| ***Cell motiluty*** | |
| *Cap1* | CAP, adenylate cyclase-associated protein 1 (yeast) |
| *Stmn1* | stathmin 1 |
| *Podxl* | podocalyxin-like |
| ***Mitochondrion*** | |
| *Grpel1* | GrpE-like 1, mitochondrial |
| *Acadm* | acyl-Coenzyme A dehydrogenase, medium chain |
| *Cox4i1* | cytochrome c oxidase subunit IV isoform 1 |
| *Acaa2* | acetyl-Coenzyme A acyltransferase 2 (mitochondrial 3-oxoacyl-Coenzyme A thiolase) |
| *Alas2* | aminolevulinic acid synthase 2, erythroid |
| *Gm2a* | GM2 ganglioside activator protein |

**Table B**

| ***Symbol*** | **Name** |
| --- | --- |
| ***Cytoplasm*** | |
| *Lyzs* | lysozyme |
| *Gsr* | glutathione reductase 1 |
| *Col4a1* | procollagen, type IV, alpha 1 |
| *Tiam2* | T-cell lymphoma invasion and metastasis 2 |
| *Apex1* | apurinic/apyrimidinic endonuclease 1 |
| *Psma4* | proteasome (prosome, macropain) subunit, alpha type 4 |
| *Hax1* | HCLS1 associated X-1 |
| *Ppp1cc* | protein phosphatase 1, catalytic subunit, gamma isoform |
| *Hmgcl* | 3-hydroxy-3-methylglutaryl-Coenzyme A lyase |
| ***Mitochondrion*** | |
| *Lyzs* | lysozyme |
| *Gsr* | glutathione reductase 1 |
| *Ppp1cc* | protein phosphatase 1, catalytic subunit, gamma isoform |
| *Hax1* | HCLS1 associated X-1 |
| *Hmgcl* | 3-hydroxy-3-methylglutaryl-Coenzyme A lyase |
| ***Cellular metabolism*** | |
| *Sox11* | SRY-box containing gene 11 |
| *Lyzs* | lysozyme |
| *Gstp1* | glutathione S-transferase, pi 1 |
| *Mcm7* | minichromosome maintenance deficient 7 (S. cerevisiae) |
| *Ash2l* | ash2 (absent, small, or homeotic)-like (Drosophila) |
| *Gsr* | glutathione reductase 1 |
| *Psma4* | proteasome (prosome, macropain) subunit, alpha type 4 |
| *Apex1* | apurinic/apyrimidinic endonuclease 1 |
| *Ppp1cc* | protein phosphatase 1, catalytic subunit, gamma isoform |
| *Zfp54* | zinc finger protein 54 |
| *Prkx* | protein kinase, X-linked |
| *Mnat1* | menage a trois 1 |
| *Pnp* | purine-nucleoside phosphorylase |
| *Prps1* | phosphoribosyl pyrophosphate synthetase 1 |
| *Scye1* | small inducible cytokine subfamily E, member 1 |
| *Adss* | adenylosuccinate synthetase, non muscle |
| ***Glutathione metabolism*** | |
| *Gstp1* | glutathione S-transferase, pi 1 |
| *Gsr* | glutathione reductase 1 |
| ***Nucleic acid metabolism*** | |
| *Sox11* | SRY-box containing gene 11 |
| *Mcm7* | minichromosome maintenance deficient 7 (S. cerevisiae) |
| *Ash2l* | ash2 (absent, small, or homeotic)-like (Drosophila) |
| *Apex1* | apurinic/apyrimidinic endonuclease 1 |
| *Zfp54* | zinc finger protein 54 |
| *Mnat1* | menage a trois 1 |
| *Pnp* | purine-nucleoside phosphorylase |
| *Scye1* | small inducible cytokine subfamily E, member 1 |
| *Adss* | adenylosuccinate synthetase, non muscle |
| *Prps1* | phosphoribosyl pyrophosphate synthetase 1 |
| ***Cell division*** | |
| *Ppp1cc* | protein phosphatase 1, catalytic subunit, gamma isoform |
| *Cdc42* | cell division cycle 42 homolog (S. cerevisiae) |
| ***Cell cycle*** | |
| *Mcm7* | minichromosome maintenance deficient 7 (S. cerevisiae) |
| *Ppp1cc* | protein phosphatase 1, catalytic subunit, gamma isoform |
| *Mnat1* | menage a trois 1 |
| *Cdk2ap1* | CDK2 (cyclin-dependent kinase 2)-associated protein 1 |
| ***Catalytic activity*** | |
| *Lyzs* | lysozyme |
| *Gstp1* | glutathione S-transferase, pi 1 |
| *Mcm7* | minichromosome maintenance deficient 7 (S. cerevisiae) |
| *Gsr* | glutathione reductase 1 |
| *Psma4* | proteasome (prosome, macropain) subunit, alpha type 4 |
| *Apex1* | apurinic/apyrimidinic endonuclease 1 |
| *Cdc42* | cell division cycle 42 homolog (S. cerevisiae) |
| *Ppp1cc* | protein phosphatase 1, catalytic subunit, gamma isoform |
| *Prkx* | protein kinase, X-linked |
| *Hmgcl* | 3-hydroxy-3-methylglutaryl-Coenzyme A lyase |
| *Pnp* | purine-nucleoside phosphorylase |
| *Scye1* | small inducible cytokine subfamily E, member 1 |
| *Adss* | adenylosuccinate synthetase, non muscle |
| *Prps1* | phosphoribosyl pyrophosphate synthetase 1 |
